# Exercise-Induced Improvements in Insulin Sensitivity Are Not Attenuated by a Family History of Type 2 Diabetes

**DOI:** 10.3389/fendo.2020.00120

**Published:** 2020-03-13

**Authors:** Manuel Amador, Cesar A. Meza, Andrew J. McAinch, George A. King, Jeffrey D. Covington, Sudip Bajpeyi

**Affiliations:** ^1^Metabolic, Nutrition and Exercise Research (MiNER) Laboratory, Department of Kinesiology, University of Texas at El Paso, El Paso, TX, United States; ^2^Institute for Health and Sport, College of Health and Biomedicine, Victoria University, Melbourne, VIC, Australia; ^3^Australian Institute for Musculoskeletal Science, Victoria University, Melbourne, VIC, Australia; ^4^Department of Pathology and Laboratory Medicine, University of Vermont, Burlington, VT, United States

**Keywords:** combined exercise, concurrent exercise, diabetes, family history of type 2 diabetes, insulin sensitivity, metabolic flexibility, Mexican-American

## Abstract

**Introduction:** A family history of type 2 diabetes (FH+) is a major risk factor for the development of insulin resistance and type 2 diabetes. However, it remains unknown whether exercise-induced improvements in insulin sensitivity and metabolic flexibility are impacted by a FH+. Therefore, we investigated whether improvements in insulin sensitivity, metabolic flexibility, body composition, aerobic fitness and muscle strength are limited by a FH+ following eight weeks of combined exercise training compared to individuals without a family history of type 2 diabetes (FH–).

**Methods:** Twenty (*n* = 10 FH–, *n* = 10 FH+) young, healthy, sedentary, normoglycemic, Mexican-American males (age: FH– 22.50 ± 0.81, FH+ 23.41 ± 0.86 years; BMI: FH– 27.91 ± 1.55, FH+ 26.64 ± 1.02 kg/m^2^) underwent eight weeks of combined aerobic and resistance exercise training three times/week (35 min aerobic followed by six full-body resistance exercises). Insulin sensitivity was assessed via hyperinsulinemic euglycemic clamps. Metabolic flexibility was assessed by the change in respiratory quotient from fasted to insulin-stimulated states. Body composition was determined using dual-energy x-ray absorptiometry. Aerobic fitness was determined by a graded exercise test, and upper- and lower-body strength were assessed via one-repetition maximum bench press and leg strength dynamometer, respectively.

**Results:** Insulin sensitivity, metabolic flexibility, aerobic fitness and strength were not different between groups (*p* > 0.05). Eight weeks of combined aerobic and resistance exercise training improved insulin sensitivity (FH– *p* = 0.02, FH+ *p* = 0.002), increased fat free mass (FH– *p* = 0.006, FH+ *p* = 0.001), aerobic fitness (FH– *p* = 0.03, FH+ *p* = 0.002), and upper- (FH– *p* = 0.0001, FH+ *p* = 0.0001) and lower-body strength (FH– *p* = 0.0009, FH+ *p* = 0.0003), but did not change metabolic flexibility (*p* > 0.05) in both groups. Exercise-induced improvements in metabolic outcomes were similar between groups.

**Conclusions:** Insulin sensitivity, metabolic flexibility, aerobic fitness and strength were not compromised by a FH+. Additionally, a FH+ is not a limiting factor for exercise-induced improvements in insulin sensitivity, aerobic fitness, body composition, and strength in normoglycemic young Mexican-American men.

## Introduction

A family history of type 2 diabetes is a major risk factor for the development of insulin resistance. The relatives of type 2 diabetes patients are 30–70% more likely to develop type 2 diabetes (1, 2) and often display metabolic aberrations including larger abdominal adipocytes (3) and lower resting energy expenditure (4). However, impairments in insulin sensitivity are not always observed in normoglycemic offspring with a family history of type 2 diabetes (FH+) compared to those without a family history of type 2 diabetes (FH–) (5–8).

There have been various hypotheses to explain type 2 diabetes heritability, suggesting both genetic (9) and environmental (10) factors; although, the complicated pathophysiology of type 2 diabetes makes it challenging to define the relative roles of modifiable and non-modifiable risk factors. A large prospective study of over 27,000 European individuals with a FH+ reported that neither lifestyle, anthropometric or genetic risk factors underpinned the risk of developing type 2 diabetes (2). Nonetheless, certain ethnic groups display a higher prevalence of type 2 diabetes. Compared with non-Hispanic Whites, Hispanics tend to be more insulin resistant (11) and among Hispanics/Latinos, Mexican-Americans have been reported to have the highest prevalence of type 2 diabetes (12). This insulin resistance in Mexican-Americans has been attributed to reduced peripheral glucose disposal, rather than impaired insulin secretion (13). Furthermore, the increased risk of developing type 2 diabetes in Hispanics with a FH+ is likely compounded by excess adiposity, as overweight Hispanic children display impaired glucose tolerance and beta-cell dysfunction (14). These data support the contention that Mexican-Americans, and particularly those with a FH+, have an increased risk of developing type 2 diabetes; however, it remains to be understood whether the insulin resistance affects improvements in glucose homeostasis associated with exercise training. In addition, it remains unclear whether metabolic flexibility is affected by a FH+ given the contradictory findings (5, 7, 15, 16).

Exercise and weight loss are considered effective strategies for type 2 diabetes treatment (17), and exercise training alone is sufficient to improve insulin sensitivity in FH+ (18). However, a recent study reported that young, healthy individuals with a FH+ were unable to suppress fat oxidation during a bout of high-intensity exercise that followed a mixed meal (19). This suggests that even young, normoglycemic offspring of type 2 diabetes patients have impaired metabolic flexibility, likely increasing the predisposition to developing metabolic disease later in life. In addition, a metabolic inflexibility may attenuate the improvements in insulin sensitivity associated with chronic exercise, although this has not been studied, to our knowledge. Only three studies (18, 20, 21) have investigated the effects of exercise training on insulin sensitivity in individuals with a FH+, and the effects of resistance training remain to be studied in this population. While resistance exercise alone elicits improvements in muscular and metabolic function (22), combining aerobic and resistance exercise can improve insulin sensitivity and metabolic flexibility in type 2 diabetes patients (16) as well as augment skeletal muscle insulin signaling (23) and reduce glycated hemoglobin (HbA1c) (24). However, there are no studies that have investigated whether a FH+ attenuates exercise-induced improvements in insulin sensitivity or metabolic flexibility in young, normoglycemic Mexican-Americans. Thus, investigating the effects of combined aerobic and resistance exercise training on insulin sensitivity in individuals predisposed to developing type 2 diabetes provides an opportunity to examine potential therapies and reduce the overall prevalence of type 2 diabetes.

Therefore, the purpose of this study was to investigate if normoglycemic, otherwise healthy, young adult Mexican-American males with a first-degree FH+ (1) are more insulin resistant or metabolically inflexible compared to individuals without a family history of type 2 diabetes and (2) are able to achieve exercise-induced improvements in insulin sensitivity, metabolic flexibility, aerobic fitness, body composition, or muscle strength to a similar extent after eight weeks of combined aerobic and resistance exercise training, compared to those without a family history of type 2 diabetes. We hypothesized that insulin sensitivity and metabolic flexibility would be lower in individuals with a FH+, and that exercised-induced improvements in insulin sensitivity and metabolic flexibility would be impeded by a FH+.

## Materials and Methods

### Participants

This study was approved by the institutional review board of the University of Texas at El Paso. All subjects provided informed consent. Subjects were recruited from the US-Mexico border region of El Paso, TX. Twenty-two normoglycemic, healthy, sedentary Mexican-American males between the ages of 18 and 40 years were enrolled in the study intervention performed at the Metabolic, Nutrition and Exercise Research (MiNER) Laboratory of the University of Texas at El Paso (ClinicalTrials.gov NCT02745613). Subjects who self-reported a FH+ from at least one parent during the screening process were assigned to the FH+ group. Subjects with <60 min per week of moderate to vigorous physical activity (MVPA) were determined sedentary via physical activity monitors (ActiGraph Corp., Pensacola, FL). Subjects were excluded from the study for evidence of diabetes or cardiovascular disease, fasting blood glucose ≥5.5 mmol/L, screening blood pressure ≥140/90 mmHg, hyperlipidemia or total cholesterol ≥6.2 mmol/L, use of drugs affecting energy metabolism or body weight, excess alcohol, drug abuse or smoking, and eating disorders. Participants were instructed to return to the MiNER Laboratory 1 week following screening for maximal aerobic capacity, upper and lower body 1 repetition maximum (1RM), and body composition assessments. A controlled diet (55% carbohydrate, 30% fat, 15% protein) was provided for 5 days prior to baseline and post-intervention assessments to eliminate the effects of diet on primary outcome measures. All subjects performed a supervised combined aerobic and resistance exercise training intervention for eight weeks, and all baseline measurements were repeated after the combined exercise training intervention. Two subjects were withdrawn from the study including one due to non-compliance to training sessions (not included in any data analyses) and one was excluded from analyses of primary outcome measures (insulin sensitivity and metabolic flexibility) due to non-compliance of exercise and dietary prescriptions during the final week of testing. Therefore, 19 subjects were considered in the analyses of insulin sensitivity and metabolic flexibility.

### Exercise Training Protocol

All exercise sessions were supervised by trained graduate students to ensure safety and compliance to exercise prescriptions. Exercise training consisted of eight weeks of combined (aerobic followed by resistance exercise) training performed 3 days per week. Aerobic exercise training comprised of 35 min of treadmill running between 55 and 75% VO_2max_ matched by heart rate via Polar heart rate monitors (Bethpage, NY). Subjects ran at 55% VO_2max_ during the first week and intensity was increased each week until 75% VO_2max_ was reached by week 6. The resistance training protocol included six strength exercises (three sets of eight repetitions) periodized from a pool of 18 exercises. Each exercise session included three upper and three lower body resistance exercises. Assessments of metabolic outcomes were obtained within ~36 h after the last exercise bout under conditions identical to baseline.

### Insulin Sensitivity

Insulin sensitivity was determined via a hyperinsulinemic euglycemic clamp as previously published (25, 26). Participants were asked to avoid eating, drinking, smoking and strenuous exercise for 10–12 h prior to arriving at the research facility. A catheter was inserted into a forearm vein for infusion of insulin and glucose, and a catheter was inserted into a contralateral dorsal hand vein warmed in a heating blanket for arterialized-venous blood sampling. Fasting insulin and glucose samples were obtained prior to insulin and glucose infusion. A primed continuous infusion of regular insulin (Humulin, Eli Lilly and Co., Indianapolis, IN) was administered at a rate of 80 mU/m^2^ body surface area per minute from 0 to 120 min together with 20% dextrose solution to maintain blood glucose at a concentration of 5.0 mmol/L throughout the clamp period. Blood samples were collected every 5 min during the clamp and immediately analyzed for determination of glucose concentrations (YSI 2300 STAT Plus, Yellow Springs, OH). Fasting plasma insulin concentrations were measured from blood collected during pre- and post-intervention clamps and analyzed by Laboratory Corporation of America (Burlington, NC). Insulin sensitivity was determined from the glucose disposal rate (mg/kg EMBS/min) obtained during the final 15 min of the clamp and normalized to estimated metabolic body size to account for differences in metabolic tissue mass between participants, as individuals with greater adiposity may have lower metabolizable tissue mass (25–27).

### Substrate Utilization and Metabolic Flexibility

Resting metabolic rate (RMR) was measured via indirect calorimetry using a Parvomedics TrueOne 2400 metabolic measurement cart (Salt Lake City, UT). On the same day as the clamp, participants' RMR and respiratory quotient (RQ) were determined by indirect calorimetry. Metabolic flexibility was determined by subtracting the fasting RQ from the insulin-stimulated RQ (ΔRQ).

### Body Composition

Body composition was assessed via dual-energy x-ray absorptiometry (GE Medical Systems. Madison, WI) the day before pre/post-intervention clamps. Measurements of fat free mass, fat mass, bone mineral density (BMD), and bone mineral content (BMC) were obtained.

### Maximal Aerobic Capacity

Maximal aerobic capacity (VO_2max_) was assessed using a standardized graded exercise treadmill test. Subjects began the test at 3 mph and a 3% incline for the first 3 min followed by a 2 min warm up at 5 mph at 0% incline. The remainder of the test was performed at a self-selected speed while the % grade was increased each minute. Subjects' rate of perceived exertion (RPE) and heart rate (HR) were monitored throughout the test. The test was terminated once the subject reached volitional exhaustion. Standard measurements of VO_2_, VCO_2_ were collected, via a Parvomedics TrueOne 2400 metabolic measurement cart (Salt Lake City UT), to determine respiratory exchange ratio (RER) continuously throughout the exercise testing. Maximal aerobic capacity was determined once the subject met two out of the four following criteria: respiratory exchange ratio (RER) >1.1, within 10 beats per minute from age predicted maximal heart rate (220—age), RPE > 17, when a plateau in oxygen consumption was achieved.

### Maximal Strength

Assessments of maximal strength were performed at baseline and during the last week of the exercise training intervention. Maximal upper and lower body strength were assessed via 1RM bench press and back leg strength dynamometer, respectively.

### Dietary Control

Subjects were provided a 5 day standardized diet (55/30/15% carbohydrate/fat/protein) prior to pre- and post-intervention testing. Meals were designed to comply with the USDA 2010 Dietary Guidelines for Americans and individualized to participant preferences. Breakfast, lunch, dinner, and snacks were weighed, prepared, and organized by meal and day to ensure compliance. The Mifflin St Jeor equation (28) was utilized to match the participants' estimated energy requirements. Participants were asked to consume an energy balanced diet according to the USDA 2010 Dietary Guidelines for Americans throughout the exercise training intervention.

### Statistical Analysis

Statistical analyses were performed using GraphPad Prism, version 6.0 (GraphPad Software Inc., La Jolla, California). Two-way ANOVA with repeated measures was used to compare groups (FH– vs. FH+), time (before vs. after intervention) and group by time effects. Significant differences were assumed for *p* < 0.05.

## Results

Table 1 summarizes the subjects' baseline characteristics and outcomes following eight weeks of combined aerobic and resistance exercise training. At baseline, age, BMI, fat free mass, fat mass, fasting glucose, fasting insulin, lipid profile, RMR, aerobic fitness, and strength were not significantly different between FH– and FH+ groups.

**Table 1:** Characteristics of subjects before and after eight weeks of combined exercise training.

	**No Family History (FH–)**		**Family History (FH+)**		**FH– vs. FH+**			
	**(*****n*** **=** **10)**		**(*****n*** **=** **10)**		**Baseline**	**Group**	**Time**	**Interaction**
	**Baseline**	**Post-intervention**	**Baseline**	**Post-intervention**	***p*-value**	***p*-value**	***p*-value**	***p*-value**
Age (years)	22.50 ± 0.81		23.41 ± 0.86		0.25			
Baseline physical activity level (PAL)	0.38 ± 0.17		0.69 ± 0.44		0.48			
**Body composition**								
Height (cm)	174.37 ± 1.31		174.12 ± 1.50		0.35			
Body weight (kg)	80.87 ± 4.62	83.69 ± 4.69*	79.00 ± 2.81	79.66 ± 2.57	0.95	0.59	**0.0004**	**0.01**
BMI (kg/m^2^)	27.91 ± 1.55	28.35 ± 1.55	26.64 ± 1.02	26.71 ± 1.01	0.50	0.43	0.14	0.27
Body fat (%)	29.30 ± 2.10	28.03 ± 2.04*	31.63 ± 2.06	30.01 ± 1.9*	0.43	0.45	**0.0004**	0.60
Fat mass (kg)	23.97 ± 2.76	23.91 ± 2.78	24.23 ± 1.97	23.21 ± 1.82	0.94	0.94	0.12	0.17
Fat free mass (kg)	55.58 ± 2.17	57.72 ± 2.06*	51.14 ± 1.58	53.42 ± 1.8*	0.11	0.12	**0.0001**	0.87
Waist-to-hip ratio (WHR)	0.89 ± 0.01	0.87 ± 0.01	0.86 ± 0.01	0.84 ± 0.01	0.33	0.39	**0.02**	0.84
Bone mineral content (g)	3089.10 ± 121.21	3111.30 ± 136.49	2944.70 ± 127.55	3033.70 ± 120.06	0.42	0.53	0.09	0.30
Bone mineral density (g/kg^2^)	1.25 ± 0.03	1.25 ± 0.03	1.23 ± 0.04	1.23 ± 0.04	0.69	0.71	0.19	0.79
**Glycemic control**								
Fasting glucose (mmol/L)^†^	4.29 ± 0.10	4.34 ± 0.07	4.47 ± 0.09	4.46 ± 0.08	0.23	0.22	0.73	0.62
Fasting insulin (mIU/L)^†^	12.9 ± 2.45	10.12 ± 1.59*	9.62±.87	9.53 ± 1.09	0.20	0.37	0.05	0.06
**Lipids**								
Total cholesterol (mmol/L)^†^	3.69 ± 0.18	3.91 ± 0.23	4.13 ± 0.33	3.95 ± 0.30	0.27	0.53	0.86	0.06
Triglycerides (mmol/L)^†^	1.72 ± 0.25	1.82 ± 0.23	1.50 ± 0.24	1.28 ± 0.23	0.53	0.25	0.50	0.09
HDL cholesterol (mmol/L)^†^	0.81 ± 0.06	0.91 ± 0.06*	0.99 ± 0.09	1.06 ± 0.09*	0.12	0.13	**0.0001**	0.69
LDL cholesterol (mmol/L)^†^	2.06 ± 0.16	2.13 ± 0.15	2.46 ± 0.29	2.29 ± 0.25	0.26	0.37	0.59	0.17
Total cholesterol/HDL ratio^†^	4.71 ± 0.35	4.42 ± 0.27	4.57 ± 0.61	4.06 ± 0.60*	0.84	0.71	**0.01**	0.44
LDL/HDL ratio^†^	2.63 ± 0.24	2.42 ± 0.19	2.77 ± 0.48	2.42 ± 0.46*	0.80	0.89	**0.01**	0.46
**Substrate utilization**								
Resting metabolic rate (Kcal/day)^†^	2077.32 ± 119.5	2073 ± 61.05	1965.97 ± 50.62	2061.40 ± 87.36	0.29	0.40	0.27	0.40
Resting metabolic rate (Kcal/FFM)^†^	37.6 ± 1.16	36.6 ± 1.00	38.6 ± 1.27	38.9 ± 1.52	0.57	0.32	0.65	0.40
Resting metabolic rate (Kcal/BW)^†^	25.2 ± 0.81	25.0 ± 1.06	24.9 ± 0.72	25.4 ± 0.81	0.82	0.96	0.80	0.56
Fasting substrate utilization (RQ)^†^	0.72 ± 0.00	0.69 ± 0.01	0.71 ± 0.01	0.72 ± 0.01	0.07	0.81	0.44	0.18

Data are presented as mean ± SEM;

† indicates n = 9 for FH– group; *significant difference compared with the respective baseline value, p < 0.05. Bold text indicates statistical significance, p < 0.05.

### Effects of Combined Exercise Training on Insulin Sensitivity and Metabolic Flexibility

**Figure 1:**
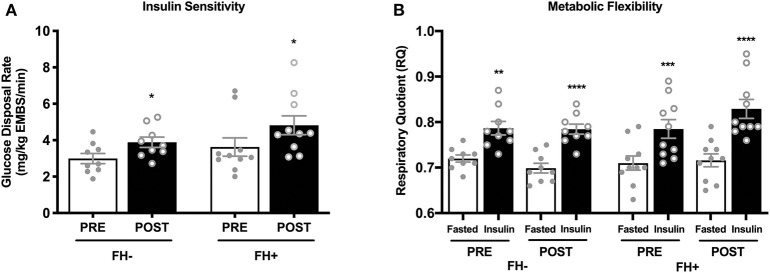
**(A)** Healthy normoglycemic individuals with (FH+) and without (FH–) a family history of type 2 diabetes significantly improved insulin sensitivity after eight weeks of combined aerobic and resistance exercise training. **(B)** Metabolic flexibility did not change in either group after eight weeks of combined aerobic and resistance exercise. Respiratory quotient significantly increased in both groups from fasting conditions to insulin stimulated conditions during pre and post intervention clamps. *p < 0.05, **p < 0.01, ***p < 0.001, ****p < 0.0001. Data are means ± SEM.

Insulin sensitivity and metabolic flexibility were similar between FH– and FH+ at baseline. Eight weeks of combined exercise training led to significant increases (*p* = 0.002) in insulin sensitivity with no differences between groups (*p* = 0.51) (FH– 2.99 ± 0.27 to 3.89 ± 0.28; FH+ 3.63 ± 0.50 to 4.82 ± 0.51 mg/kg EMBS/min) (Figure 1A). Improvements in insulin sensitivity in response to exercise training, expressed as delta (Δ) (FH– 0.89 ± 0.25 vs. FH+ 1.19 ± 0.35) or percent (%) change (FH– 34.90 ± 11.0% vs. FH+ 40.66 ± 12.19%), were comparable regardless of family history status. Metabolic flexibility was unaltered (*p* = 0.12) after eight weeks of combined exercise training in both FH– (0.07 ± 0.01 to 0.09 ± 0.02 ΔRQ) and FH+ (0.08 ± 0.01 to 0.11 ± 0.02 ΔRQ) groups (Figure 1B). There were no changes in RMR, and fasting RQ following the exercise intervention and were not different between groups (Table 1).

### Effects of Combined Exercise Training on Substrate Utilization and Metabolic Markers

There were no differences in baseline RMR (FH– 2077 ± 119.50 vs. FH+ 1966 ± 50.62 kcal/day) or RQ (FH– 0.72 ± 0.01 vs. FH+ 0.71 ± 0.01 RQ) between FH– and FH+, and were unaltered by eight weeks of combined exercise training (Table 1). The levels of plasma triglycerides, total cholesterol and low-density lipoprotein (LDL) cholesterol were similar between groups at baseline and unaffected by the exercise intervention (Table 1). High-density lipoprotein (HDL) cholesterol was similar between groups at baseline and significantly increased following the exercise intervention in FH– only (Table 1). The ratios of total cholesterol to HDL and LDL to HDL significantly improved in the FH+ group only following the exercise intervention (Table 1).

### Effects of Combined Exercise Training on Body Composition, Aerobic Fitness and Strength

**Figure 2:**
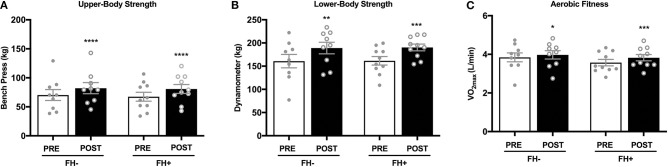
**(A)** Healthy normoglycemic individuals with (FH+) and without (FH–) a family history of type 2 diabetes significantly increased upper body strength after eight weeks of combined aerobic and resistance exercise training. **(B)** Individuals with (FH+) and without (FH–) a family history of type 2 diabetes significantly increased lower body strength. **(C)** Individuals with (FH+) and without (FH–) a family history of type 2 diabetes improved aerobic fitness after eight weeks of combined aerobic and resistance exercise training. *p < 0.05, **p < 0.01, ***p < 0.001, ****p < 0.0001. Data are means ± SEM.

**Figure 3:**
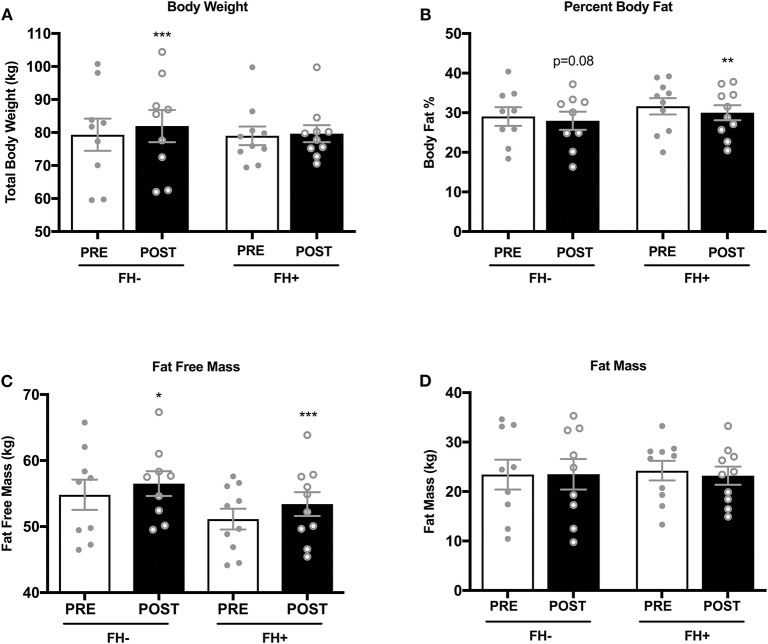
**(A)** Healthy normoglycemic individuals without (FH–) a family history of type 2 diabetes significantly increased total body weight after eight weeks of combined aerobic and resistance exercise training. **(B)** Percent body fat significantly decreased in individuals with a family history of type 2 diabetes (FH+). **(C)** Both FH– and FH+ significantly increased fat free mass **(D)** No changes in fat mass was observed in either group. *p < 0.05, **p < 0.01, ***p < 0.001. Data are means ± SEM.

Upper (Figure 2A) and lower body (Figure 2B) strength significantly increased in both groups (upper body FH– 70.31 ± 9.45 to 82.15 ± 9.58 kg; FH+ 67.36 ± 7.65 to 80.74 ± 7.65 kg; lower body FH– 160.77 ± 14.50 to 189.00 ± 12.50 kg; FH+ 161.48 ± 9.15 to 190.28 ± 7.25 kg) following eight weeks of combined exercise training (Figures 2A,B). The FH+ group significantly improved maximal aerobic capacity expressed as relative (ml/kg/min) and absolute (L/min) fitness, however FH– only improved absolute VO_2max_ (Figure 2C). Body composition (body weight, fat mass, fat free mass) was similar between groups at baseline (Table 1). Body weight significantly increased in FH– only following the exercise intervention (Figure 3A). There were significant reductions in percent body fat and increases in fat free mass and no change in fat mass in both groups after eight weeks of combined exercise training (Figures 3B–D). There were no changes in waist-to-hip ratio (WHR) regardless of FH (Table 1).

## Discussion

A family history of type 2 diabetes is considered a major risk factor for the development of insulin resistance, and Mexican-Americans, in particular, are at a greater risk for developing type 2 diabetes compared with non-Hispanic whites (29). In this study of young Mexican-Americans with an increased predisposition for type 2 diabetes, we report the effects of eight weeks of combined aerobic and resistance exercise training on insulin sensitivity and metabolic flexibility. The primary findings from this study are that exercise-induced improvements in insulin sensitivity, body composition, aerobic fitness and muscle strength are not attenuated by a family history of type 2 diabetes in young, healthy, normoglycemic Mexican-American men following an eight week combined exercise training intervention. Others have reported that aerobic exercise training is an effective strategy to improve insulin sensitivity in Caucasians with a FH+ (18, 20, 21) and exercise can improve insulin sensitivity in Hispanics predisposed to type 2 diabetes (30) and with overt type 2 diabetes (23). However, we are the first to report that the addition of resistance exercise to aerobic exercise training enhances insulin sensitivity and physical function, as shown by concomitant improvements in upper- and lower-body strength, as well as increased aerobic capacity.

Interventions that target the relatives of type 2 diabetes patients can provide important insight related to the modifiable and non-modifiable risk factors that contribute to metabolic disease. Our study indicates that a FH+ does not diminish insulin sensitivity in young, normoglycemic Mexican-Americans. These findings are in accordance with previous reports in non-Hispanic populations (5, 6); however, others have reported that Mexican-Americans with a FH+ display insulin resistance (15, 31–33). Mexican-Americans with a FH+ have been characterized by an array of metabolic defects, including impairments in non-oxidative glucose disposal and suppression of lipid oxidation during hyperinsulinemic euglycemic clamps (15). Bonora et al. (32) indicated that, compared with non-Hispanic White women, Mexican-American women with a FH+ are more insulin resistant. Similarly, there is reduced insulin-stimulated glucose disposal in Mexican-Americans (31) and a cohort of both Mexican-American and non-Hispanic white adults with a FH+ (33). However, it should be noted that the mean age (22 years) of the subjects in our study is lower compared with the subjects' ages in the studies mentioned above (38–43 years). This distinction in age may explain the absence of insulin resistance in our study population, as it has been reported that the combined prevalence of diabetes, hyperglycemia, and impaired glucose tolerance increased with age from 20.9% (20–39 years) to 46.9% (40–59 years) in U.S. adults (34). Middle-aged individuals with a FH+ are also 40–50% more likely to develop prediabetes compared with FH– counterparts (35, 36). Therefore, our findings of normal insulin sensitivity despite a FH+ suggest that the prevention of type 2 diabetes requires intervention during the progression from young to middle age, a time course that may change the FH– classification as parents would have a greater chance to develop type 2 diabetes. Given that insulin resistance is often the earliest detectable impairment in glucose homeostasis before the development of type 2 diabetes (13, 37), results from the present study have important implications for reducing the burden of this disease. Future studies are warranted to determine whether a FH+ accelerates the age-related declines in insulin sensitivity.

The current study highlights that exercise training-induced improvements in insulin sensitivity were not attenuated by the presence of a FH+. Our findings of normal insulin sensitivity and similar magnitudes of improvement between groups, suggest the absence of skeletal muscle insulin resistance despite a FH+. The enhancements of insulin-stimulated glucose disposal can persist 48 h after a single exercise session (21), and chronic exercise training augments the exercise-related adaptations in muscle glucose uptake (38). Thus, participation in long-term exercise training abrogate the reductions in insulin sensitivity observed in certain populations with a FH+. The present study also demonstrated concurrent improvements in VO_2max_, lean mass, and upper and lower body strength after eight weeks of combined exercise training. Combining aerobic and resistance training in men and women with type 2 diabetes elicits improvements in fasting and postprandial glucose concentrations (23, 24), increases in skeletal muscle insulin receptor substrate 1 (IRS-1) protein content (23), and improvements in muscular strength and hypertrophy (24). Both the FH– and FH+ groups in our study displayed improvements in upper body (FH– increased by 19%; FH+ increased by 22%) and lower body strength (FH– increased by 22%; FH+ increased by 19%) as well as significant increases in fat free mass. Resistance and aerobic training in the current study elicited improvements in aerobic capacity, muscular strength and hypertrophy, in addition to insulin sensitivity. These results indicate that individuals predisposed to type 2 diabetes due to a FH+ should undertake combined exercise training to delay or prevent insulin resistance.

Metabolic flexibility refers to the ability to adapt fuel oxidation to fuel availability (39). Individuals with obesity and with type 2 diabetes have a blunted shift toward carbohydrate oxidation during maximal exercise as well as an impaired suppression of lipid oxidation (i.e., metabolic inflexibility) during hyperinsulinemia (40). While, exercise training can elicit a complete restoration of metabolic flexibility in type 2 diabetes patients (16), the present study shows that metabolic flexibility was not affected by a FH+ and was unaltered by eight weeks of combined aerobic and resistance exercise training. The rates of whole-body fat oxidation detected at baseline in our study (RQ: ~0.70) indicated that basal substrate metabolism was already at maximal capacity in both groups, and insulin-stimulation during the clamp did not blunt the increases in carbohydrate oxidation. Our findings of increased insulin sensitivity without changes in metabolic flexibility suggest that the improvements in insulin-stimulated glucose disposal were primarily non-oxidative, and can be explained by enhanced insulin-stimulated muscle glycogen synthesis. This is in accordance with a previous report showing that aerobic exercise improves insulin-stimulated glucose disposal in a non-oxidative manner (21). In the study by Perseghin et al. (21), insulin resistant subjects with a FH+ displayed lower muscle glucose-6-phosphate concentrations and rates of glycogen synthesis, and despite the increases after 6 weeks of aerobic exercise training, glycogen synthesis remained lower in FH+ compared with FH–. Therefore, defects in hexokinase and glycogen synthase activity appear to be early markers of insulin resistance in individuals with a FH+, a contention that has been supported by others (41, 42). It should be noted that glycogen synthesis was not measured in the present study; however, it is likely that the improvements in insulin sensitivity following eight weeks of combined exercise training were due to increased non-oxidative glucose disposal considering that carbohydrate oxidation during the clamp was unaltered. Similarly, a FH+ does not blunt the improvements in VO_2max_ following aerobic exercise training alone (20) or combined aerobic and resistance exercise training (Figure 2C). Thus, it appears the reductions in maximal aerobic capacity observed in type 2 diabetes are not present in healthy young men with a FH+, and the ability to improve aerobic capacity with exercise training is not attenuated. This is important for Mexican-Americans and those considered at-risk for insulin resistance, as better cardiorespiratory fitness has been linked with lower risk of all-cause mortality (43).

One of the strengths of this study is the use of the hyperinsulinemic euglycemic clamp technique to assess insulin sensitivity. The present study is novel in the addition of resistance exercise to an exercise program, and all exercise sessions were supervised and standardized to include aerobic exercise followed by resistance exercise during each training session. In addition, the dietary control of the 5 day period prior to assessment of insulin sensitivity was used minimize the effects of dietary variability on primary outcome measures, although we did not control for energy intake throughout the exercise training intervention or collect nutritional intake data using a dietary recall. Limitations of this study include the relatively small sample size, the lack physical activity data throughout the exercise training intervention, the short duration of the exercise training intervention and the lack of exercise groups that only performed aerobic or resistance exercise training. Therefore, we cannot compare the effectiveness of combined exercise training on insulin sensitivity to aerobic or resistance exercise training alone. Additionally, we attest that while whole body glucose disposal rates are a strong indication of skeletal muscle insulin sensitivity, we did not determine adipose or liver insulin sensitivity through the use of tracers or determine plasma insulin and free fatty acid concentrations throughout the clamps. Lastly, the findings from this study are limited to Mexican-American males and the relative contributions of a FH+ on insulin sensitivity between males and females and in other ethnic groups remain unclear.

In summary, we show that regardless of a FH+, individuals are not predestined to develop type 2 diabetes and thus interventions such as the current study can play a significant role in decreasing the burden of this disease in at-risk populations. A family history of type 2 diabetes does not attenuate exercise-induced improvements in insulin sensitivity in young healthy Mexican-Americans. We also demonstrate that improvements in aerobic capacity following eight weeks of combined aerobic and resistance exercise training occur in parallel with improvements in muscle strength and body composition. These findings highlight the importance of promoting aerobic and resistance exercise training as a method of preventing type 2 diabetes. Interventions in populations with a FH+ hold significant potential for the prevention of metabolic disease and warrant further investigations to determine whether combined aerobic and resistance exercise training yields similar results in older adults with a FH+, and whether long-term exercise training ultimately decreases the risk of developing type 2 diabetes later in life.

## Data Availability Statement

The datasets generated for this study are available on request to the corresponding author.

## Ethics Statement

The studies involving human participants were reviewed and approved by University of Texas at El Paso Internal Review Board (IRB). The patients/participants provided their written informed consent to participate in this study.

## Author Contributions

SB designed the study. SB, MA, and CM collected the data, analyzed the data, and wrote the manuscript. SB, MA, CM, AM, JC, and GK reviewed and revised the manuscript. SB is the guarantor of this work, and as such, had full access to all the data in the study and takes full responsibility for the integrity of the data and the accuracy of the data analysis.

### Conflict of Interest

The authors declare that the research was conducted in the absence of any commercial or financial relationships that could be construed as a potential conflict of interest.
